# Arsenic trioxide inhibits glioma cell growth through induction of telomerase displacement and telomere dysfunction

**DOI:** 10.18632/oncotarget.7259

**Published:** 2016-02-08

**Authors:** Ye Cheng, Yunqian Li, Chengyuan Ma, Yang Song, Haiyang Xu, Hongquan Yu, Songbai Xu, Qingchun Mu, Haisong Li, Yong Chen, Gang Zhao

**Affiliations:** ^1^ Department of Neurosurgery, First Hospital of Jilin University, Changchun, P. R. China

**Keywords:** As_2_O_3_, telomere, telomerase, glioma, growth inhibition

## Abstract

Glioblastomas are resistant to many kinds of treatment, including chemotherapy, radiation and other adjuvant therapies. As_2_O_3_ reportedly induces ROS generation in cells, suggesting it may be able to induce telomerase suppression and telomere dysfunction in glioblastoma cells. We show here that As_2_O_3_ induces ROS generation as well as telomerase phosphorylation in U87, U251, SHG4 and C6 glioma cells. It also induces translocation of telomerase from the nucleus to the cytoplasm, thereby decreasing total telomerase activity. These effects of As_2_O_3_ trigger an extensive DNA damage response at the telomere, which includes up-regulation of ATM, ATR, 53BP1, γ-H_2_AX and Mer11, in parallel with telomere fusion and 3′-overhang degradation. This ultimately results in induction of p53- and p21-mediated cell apoptosis, G2/M cell cycle arrest and cellular senescence. These results provide new insight into the antitumor effects of As_2_O_3_ and can perhaps contribute to solving the problem of glioblastoma treatment resistance.

## INTRODUCTION

Glioblastoma is one of the most common and devastating primary malignant intracranial tumors occurring in humans. The current therapy for newly diagnosed glioblastoma is surgical resection followed by radiotherapy plus chemotherapy [1]. However, the prognosis is poor, with a median overall survival of only 14.6 months, a median progression-free survival of 6.9 months, and a 5-year survival rate of only 9.8% after diagnosis [1, 2]. Malignant gliomas are resistant to many kinds of treatment, including chemotherapy, radiation and other adjuvant therapies. Moreover, glioma cells are prone to acquiring drug resistance systems. Consequently, there is a need to identify chemotherapeutic agents with cytotoxicity toward glioma cells [3].

Arsenic trioxide (As_2_O_3_) is a naturally occurring arsenic compound traditionally regarded as poisonous [4], though it has been used as a therapeutic agent since 15th century. In 1970s, As_2_O_3_ was found to be effective in the treatment of acute promyelocytic leukemia (APL) [5, 6], and has been tested in clinical trials of APL patients worldwide since then. There are now studies reporting the cytotoxic potential of As_2_O_3_ in many malignant tumors, including breast and lung cancers [7, 8]. In the 2000s, As_2_O_3_ was reported to inhibit growth of malignant glioma cell lines and to induce cell death. Moreover, anticancer therapy using As_2_O_3_ has been shown to be safe and effective in both the short-term and long-term [9].

The mechanism by which As_2_O_3_ induces cell death is not fully understood. The compound reportedly induces DNA and chromosomal damage, inhibits DNA repair, and alters DNA methylation in mammalian cells [10]. Phatak [11] reported that telomere erosion and reduced telomerase activity is the main cause of As_2_O_3_-induced cell toxicity. Although it is not universal, elevated telomerase activity is frequently detected in advanced cancer cells and is important for continuous cancer cell proliferation [12, 13, 14]. In glioblastoma cells, for example, over-expressed telomerase stabilizes telomeres [15]. However, there is as yet no evidence that the anti-proliferative effect As_2_O_3_ on glioblastoma cells reflects interference with telomeres or telomerase activity. Our aim in the present study was to determine the mechanism by which As_2_O_3_ might inhibit telomerase activity and the site of any induced DNA damage. We also sought to shed light on the effect of As_2_O_3_ to cell apoptosis, cell cycle arrest and cellular senescence.

## RESULTS

### As_2_O_3_ is cytotoxic and induces ROS generation in glioma cells and inhibits cell migration and invasion

**Figure 1:**
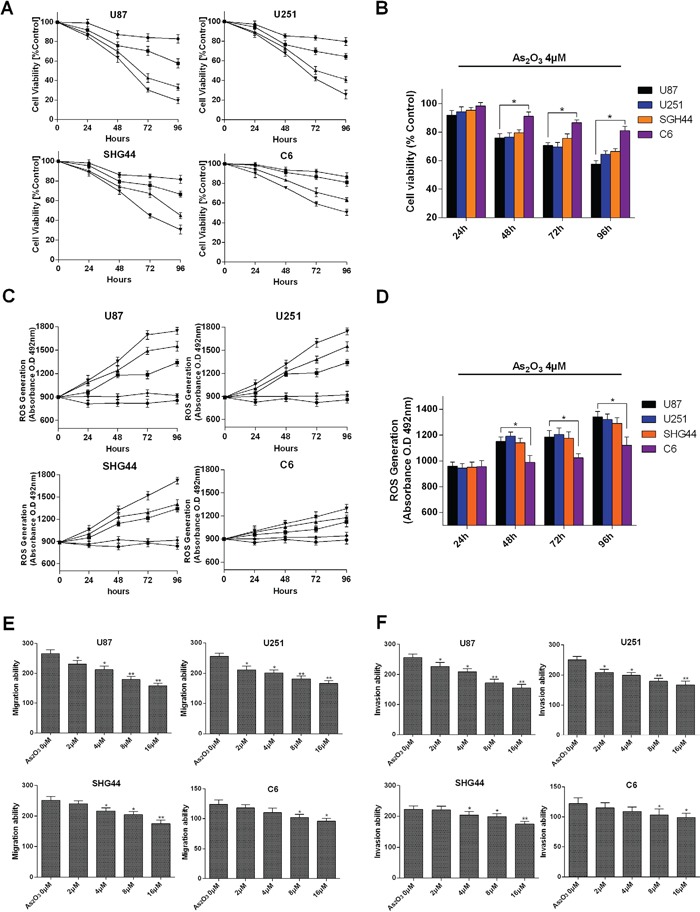
Growth suppression and ROS generation induced by As_2_O_3_ in glioma cells **A.** MTT analysis of cell viability in U87, U251, SHG44 and C6 cell lines after exposure to As_2_O_3_ at 2 (●), 4 (■), 8 (▲) or 16 μM (▼) for 24, 48, 72 and 96 h. **B.** Comparison of the viability of the indicated cells after exposure to 4 μM As_2_O_3_ for 24, 48, 72 and 96 h. **C.** ROS generation in the indicated cell lines induced by As_2_O_3_ at 0 (♦), 2 (●), 4 (■), 8 (▲) and 16 μM (▼). ROS were detected based on O.D. 492 nm measured using a microplate reader. **D.** Comparison ofROS generation in the indicate cell types after exposure to 4 μM As_2_O_3_ for 24, 48, 72 and 96 h. **E.** As_2_O_3_ inhibits migration of glioma cells *in vitro*. Migration by the indicated cell types was inhibited by pretreatment with 0-16 μM As_2_O_3_. **F.** As_2_O_3_ inhibits invasion by glioma cells *in vitro*. Error bars indicate s.d. **P<0.05, **P < 0.01, two-tailed Student's *t*-test.

We examined effect of As_2_O_3_ on the proliferation of U87, U251, SHG44 and C6 cells using MTT assays at clinically achievable As_2_O_3_ concentrations [3]. Obvious dose-and time-dependent inhibition of growth was observed in all four cell types (Figure 1A). Following exposure with As_2_O_3_ for 48 h, the 50% inhibition of growth concentrations (IC50s) were 4.45 μM in U87, 4.67 μM in U251, 4.98 μM in SHG44 and 5.56 μM in C6 cells. In all four cell types, we observed a stronger inhibitory effect at 48 h than 24 h, and the inhibitory effect was stronger at 72 h than 48 h with higher As_2_O_3_ concentrations (8 μM and 16 μM). These results are similar to those of Wu [16], who studied the time-dependent effect of As_2_O_3_ on U87 and U251 cell viability. Our study also indicates that after 48 h of treatment, the inhibitory effects significantly differ between 2 μM and 16 μM As_2_O_3_ which is similar to the finding of Wang [17], who studied the dose-dependent effect of As_2_O_3_ in U87 cells. We extended those findings by adding the study of SHG44 (another type of malignant human glioma) and C6 (mouse glioma cells) cell. Our results indicate the inhibitory effect of As_2_O_3_ is significantly weaker in C6 cells (Figure 1B), which may reflect its lower malignancy as compared to U87 and U251 cells [18]. Furthermore, we found that As_2_O_3_ induces dose-dependent generation of ROS in U87, U251 and SHG44 cells (Figure 1C). After 48 h of As_2_O_3_ treatment, the ROS generation was higher in U87 than C6 cells (Figure 1D). In addition, As_2_O_3_ significantly and dose-dependently reduced migration and invasion by U87, U251 and SHG44 cells (Figure 1E, 1F).

### As_2_O_3_ inhibits telomerase displacement, phosphorylation and activity

**Figure 2:**
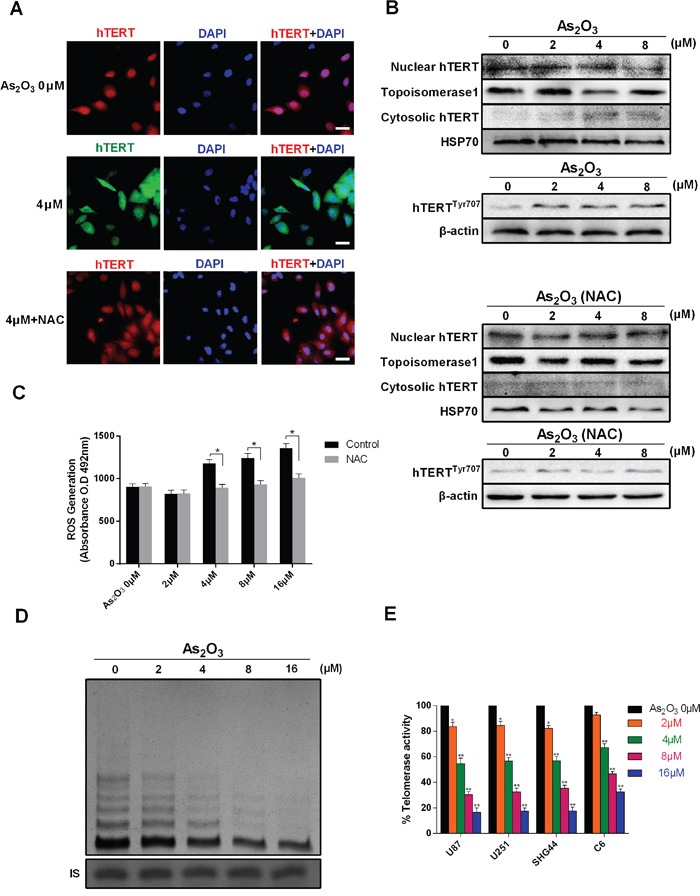
As_2_O_3_ inhibits telomerase translocation, phosphorylation and activity **A.** Immunofluorescent detection of the telomerase catalytic subunit (hTERT) in control U87 cells (green) and cells exposed to 4 μM As_2_O_3_ (green) or 4 μM As_2_O_3_ plus NAC (red). The translocation of hTERT from the nucleus to the cytoplasm was evidenced from the difference in hTERT accumulation. Cells were pretreated with NAC (1 mM) before As_2_O_3_ treatment. Scale bar = 25 μm. **B.** ROS generation induced by 0-16 μM As_2_O_3_ with and without NAC. *P < 0.05, **P < 0.01. **C.** Immunoblots showing the presence of hTERT in both nuclear and cytosolic extracts from cells exposed to 0-8 μM As_2_O_3_. The level of hTERT protein in the nucleus decreased as the cytoplasmic level increased. HSP70 was used as an internal standard for the cytoplasm, and topoisomerase 1 was used as an internal standard for the nucleus. The expression of hTERT^Tyr707^ indicated the phosphorylation of telomerase. **D.** As_2_O_3_-induced inhibition of telomerase activity in U87 cells. TRAP assays performed after 48 h of As_2_O_3_ treatment show dose-dependent inhibition of telomerase activity. The position of the internal standard was indicated as IS. **E.** Dose-dependent inhibition of telomerase activity in U87, U251, SHG44 and C6 cells. *P < 0.05, **P < 0.01 as compared with controls.

Telomerase displacement from the nucleus to the cytoplasm was examined using both immunofluorescence and immunoblotting. Immunofluorescence indicated that after treatment with 4 μM As_2_O_3_ for 48 h, there was significant cytoplasmic accumulation of telomerase catalytic subunit (hTERT), and this effect could be inhibited by NAC, a ROS scavenger (Figure 2A, 2B). This finding was confirmed by immunoblotting hTERT in both nuclear and cytosolic extract (Figure 2C, 2D). Furthermore, we found that displacement of hTERT is also dose-dependent, which is consistent with the level of ROS generation.

The detection of phosphorylated hTERT suggested that As_2_O_3_ induces Tyr707 phosphorylation of telomerase (Figure 2C). To assess the effect of As_2_O_3_ on telomerase enzymatic activity, we performed telomeric repeat amplification protocol (TRAP) assays with telomerase extracts from U87, U251, SHG44 and C6 cells. Activity levels were then determined through gray scale analysis. We found that As_2_O_3_ inducedsignificant dose- and time-dependent inhibition of telomerase activity in all four cell types, though the inhibitory effects differed significantly between U87 and C6 cells (Figure 2D, 2E and Sup Figure S1).

### As_2_O_3_ induces DNA damage and telomere instability

**Figure 3:**
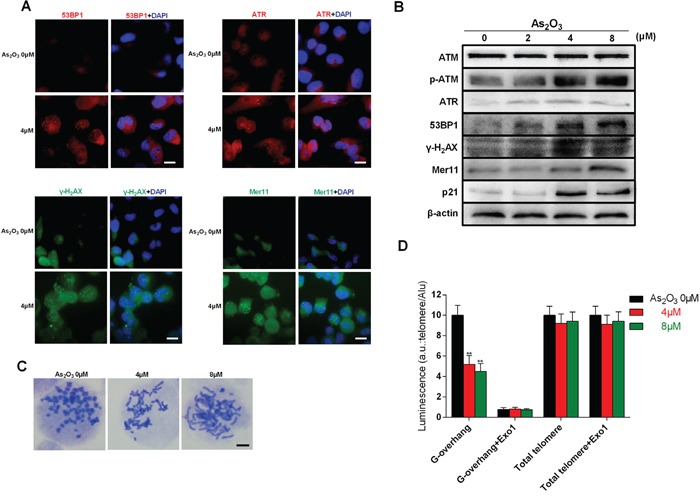
As_2_O_3_-induced DNA damage and chromosome instability is associated with degradation of telomeric G-overhang **A.** Representative immunofluorescence images of ATR, 53BP1, γ-H_2_AX and Mer11 foci in U87 cells treated with 4 μM As_2_O_3_ for 48 h. Scale bar = 15 μm. **B.** Immunoblots showing up-regulation of p-ATM, ATR, 53BP1, γ-H_2_AX, p21 and Mer11 proteins in U87 cells treated for 48 h with 4μM As_2_O_3_. Immunoblotting β-actin confirmed equivalent protein loading. Each experiment was repeated three times. **C.** Telomere fusion induced by treatment with 4 μM As_2_O_3_ for 48 h. Chromosomes were stained with Giemsa. Scale bar = 5 μm. **D.** Hybridization protection assays (HPAs) were performed on genomic DNA isolated from glioma cells treated with As_2_O_3_ to assess G-overhang length and total telomere length. *Exo 1* nuclease digestion was used to assess integrity of the 3′-overhang. Luminescence intensity in arbitrary units (AU) was normalized against Alu probe. The mean of three independent experiments with comparable results is shown. Error bars indicate ± s.d., **P < 0.01, two-tailed Student's *t*-test.

Telomerase inhibition leads to DNA damage and telomere dysfunction. Using immunofluorescence and immunoblotting, we detected DNA damage-related proteins after treatment with As_2_O_3_ in U87 cells. Immunofluorescent labeling showed that ATR, 53BP1, γ-H_2_AX and Mer11 accumulated in the nucleus of cells exposed to 4 μM As_2_O_3_ for 48h (Figure 3A). In addition, obvious dose-related increases in p-ATM, ATR, γ-H_2_AX, 53BP1, Mer11, and p21 were detected by immunoblotting (Figure 3B). This indicates a strong and complex effect of DNA damage induced by As_2_O_3_. Telomere fusion was found after exposure to As_2_O_3_ (Figure 3C). We also used hybridization protection assays (HPA) to investigate the effect of As_2_O_3_ on telomeric G-overhang length and the total telomere length. As shown in (Figure 3D), As_2_O_3_ significantly reduced the telomeric G-overhang length after 48 h of treatment (P < 0.01), though the total telomere length did not change.

### DNA-damage response triggered by As_2_O_3_ occurred at the telomere

**Figure 4:**
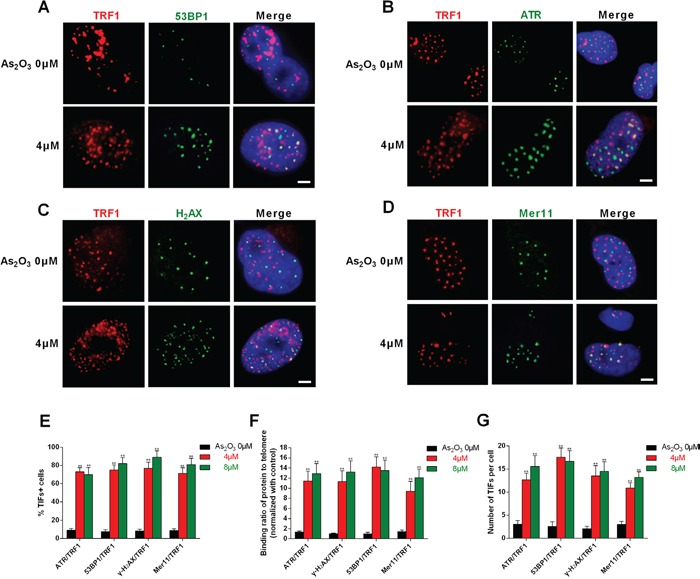
DNA-damage response triggered by As_2_O_3_ occurred at telomeres **A-D.** As_2_O_3_-treated U87 cells were double stained with the indicated antibodies. Representative confocal images showing merged TRF1 (red) with ATR, 53BP1, γ-H_2_AX or Mer11 (green) staining in untreated and As_2_O_3_-treated cells. Scale bar = 5 μm. **E.** TIF indexes, defined as foci of DNA-damage response factors that coincide with TRF1, were calculated as the percentage of TIF-positive cells among glioma cells treated with As_2_O_3_. Cells with four or more co-localization foci were scored as TIF-positive. The mean of three independent experiments was reported. Error bars indicate s.d. **P < 0.001, two-tailed Student's *t*-test. **F.** Average number of TIFs per nucleus in As_2_O_3_-treated glioma cells. The mean of three independent experiments with comparable results is shown. Error bars indicate ± s.d. **P < 0.005, two-tailed Student's *t*-test. **G.** Chip assays showed the effect of 4 μm As_2_O_3_ on binding of ATR, γ-H_2_AX, 53BP1 or Mer11 to telomeres. Data depict triplicate ChIP experiments, each with technical triplicates of qRT-PCR; **P < 0.01 as compared to control.

To verify whether ATR, γ-H_2_AX, 53BP1, and Mer11 were activated at telomeres, double immunofluorescence experiments were performed using U87 cells. Confocal microscopy revealed that most ATR, γ-H_2_AX, 53BP1 and Mer11 foci induced by As_2_O_3_ co-localized with TRF1 (Figure 4A-4D), forming so-called telomere dysfunction-induced foci (TIFs) [19]. Quantitative analysis indicated that As_2_O_3_ significantly increased the percentage of cells with more than four ATR/TRF1, γ-H_2_AX/TRF1, 53BP1/TRF1 and Mer11/TRF1 co-localizations (the percentage of TIF-positive cells reached about 65% after treatment; P < 0.01), with a mean of about eight TIFs per nucleus (Figure 4E-4F). ChIP assays confirmed that γ-H_2_AX and 53BP1 associated with telomeres in As_2_O_3_-treated cells (Figure 4G) [12, 13, 20].

### As_2_O_3_ evokes cell apoptosis, cell cycle arrest and cellular senescence

**Figure 5:**
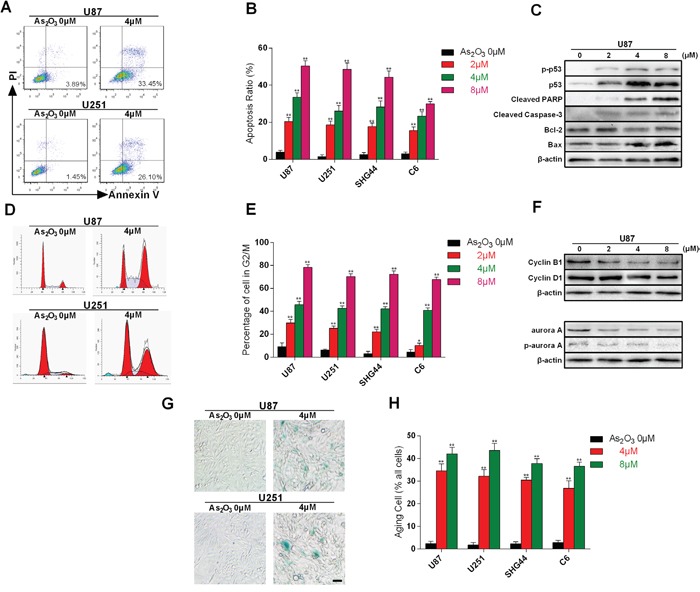
Cell apoptosis, cell cycle arrest and cellular senescence evoked by As_2_O_3_-induced telomere dysfunction **A.** Flow cytometric analysis of As_2_O_3_-induced apoptotic cell death revealed by staining with PI and Annexin V-FITC. **B.** Dose-dependence of As_2_O_3_-induced apoptosis in U87, U251, SHG4 and C6 cells. This experiment was repeated three times. **P < 0.001. **C.** Immunoblots showing the expression levels of apoptosis-related proteins. Note the dose-dependent up-regulation of p53, p-p53, Bax, cleaved PARP and cleaved Caspase-3 and down-regulation of Bcl-2 in As_2_O_3_-treated cells. **D.** Representative As_2_O_3_-induced cell cycle arrest. Cells were collected and stained with PI; DNA content was determined by flow cytometry. The percentages of cells undergoing apoptosis (sub-G1%) are expressed with respect to the total number of cells. **E.** Dose-dependent As_2_O_3_-induced G_2_/M arrest in U87, U251, SHG4 and C6 cells. This experiment was repeated three times. *P < 0.05, **P < 0.01. **F.** Immunoblots showing the expression levels of cell cycle-related proteins. **G)** Representative photomicrographs showing cellular senescence induced by exposure to As_2_O_3_ for 2 weeks. The cells are stained with β-galactosidase (SA-β-gal) stain. **H.** Dose-dependent increases in the incidence of As_2_O_3_-induced cellular senescence in U87, U251, SHG4 and C6 cells. This experiment was repeated three times. **P < 0.001.

We also explored whether As_2_O_3_-induced DNA damage in telomeres led to apoptosis, cell cycle arrest or cellular senescence. We first tested the effect of As_2_O_3_ on the incidence of apoptosis by staining cells with Annexin V and PI. As that the proportion of apoptotic cells in the lower right quadrant was increased in a dose-dependent manner (Figure 5A-5B), which is in agreement with the findings by others, who showed that As_2_O_3_ has the potential to induce apoptotic cell death [21]. Moreover, immunoblotting revealed dose- and time-dependent up-regulation of the pro-apoptotic proteins p53, p-p53 and Bax and down-regulation of anti-apoptotic protein Bcl-2 in U87 cells. We also observed elevation of PARP and Caspase-3 cleavage, which is consistent with increased incidence of apoptosis (Figure 5C). Collectively, these data suggest that glioma cells exposed to As_2_O_3_ in the short term undergo p53- and caspase-based apoptosis. This result is in agreement with that of Qian [22].

We next analyzed the percentages of cells in the different phases of the cell cycle. As shown in Figure 5D-5E, most of the cells in the control group are in G0-G1 phase. By contrast, As_2_O_3_ induced G2-M phase arrest, which is in agreement with the reported effect of As_2_O_3_ on Burkitt's lymphoma cells [23]. To provide additional evidence to support the As_2_O_3_-induced G2-M phase arrest, we used immunoblotting to detect expression of Cyclin B1, Cyclin D1, Aurora A and phospho-Aurora A. We found that As_2_O_3_ treatment led to a dose-dependent decrease of Cyclin B1, Aurora A and phospho-Aurora A, which is indicative of failure of G2 to M phase transition [24, 25]. On the other hand, the lack of change in Cyclin D1 indicates no obstruction in G1 to S transition (Figure 5F) [26].

Using aging staining we also observed that As_2_O_3_ treatment for 2 weeks increased the incidence of cellular senescence marked by cell swelling and blue staining. Dose-related effects were seen with all of four cell types tested (Figure 5G, 5H).

## DISCUSSION

Telomeres are specialized DNA structures located at the ends of chromosomes and are progressively shortened during each cell division [27]. Telomerase enables cells to escape this proliferation barrier by stabilizing telomeres [28]. It has been reported that telomerase, and specifically its catalytic subunit hTERT, is overactive in 85-90% of cancers (including glioblastomas), and it has become a widely accepted tumor marker and a popular target for anticancer therapeutics [29–30]. Telomerase inhibition can therefore be used as a therapeutic strategy for selectively targeting malignant gliomas. Usually, telomerase inhibition is accomplished through mRNA interference, expression control, phosphorylation of hTERT, or assembly and export from the nucleus [31]. Multiple kinase and phosphatase activators and inhibitors affect telomerase phosphorylation status and in turn its structure, localization and enzyme activity [32, 33]. In our study, we first found that As_2_O_3_-induced telomerase phosphorylation led to its translocation from the nucleus to the cytoplasm. Dose- and time-dependent ROS generation appears to be the main cause of hTERT phosphorylation and displacement [34]. The phosphorylation and displacement of hTERT disrupted the subunit's ability to catalyze repair of the telomere, which would lead to telomere dysfunction [35].

Telomere dysfunction can also be the result of DNA damage. DNA damage can be related to cell activities, such as malignant transformation and cell death [36]. It was recently reported that ROS is an important cause of DNA damage [37]. Thus as a ROS generator, As_2_O_3_ has the ability to induce DNA damage. However, the site(s) at which damage occurs and the mechanism remains unclear. Our study indicated that DNA damage induced by As_2_O_3_ reflects the activation of ATM and its downstream effects. The increase of phospho-ATM and ATR indicates the induction of DNA double-strand breaks as well as replication fork arrest [38]. The double-strand breaks promote expression of ATM, while replication fork blockage promotes ATR expression [39]. The increase in H_2_AX, which is induced by phospho-ATM and ATR, is part of the downstream DNA damage response. The up-regulation of 53BP1, which is also indicative of DNA double-streand breaks, is caused by the activation of ATM and ATR [40]. The up-regulation of Mer11 is another downstream effector of ATM and ATR induced in response to DNA damage. Notably, we found that ATR, 53BP1, γ-H_2_AX and Mer11 were situated on TRF1, which means the site of the DNA damage is the telomere. Previous reports indicate that arsenic has the ability to bind to the telomere at a specific binding site [41]. Our findings are consistent with that earlier result. It was also previously observed that increased ROS generation is the main reason for As_2_O_3_-induced DNA damage [42]. Consistent with that finding, we observed that As_2_O_3_ dose-dependently induced ROS generation, which led to dose-dependent increases in the expression of DNA damage proteins.

There are two primary responses after DNA double-strand breaks: DNA repair or cell cycle arrest and apoptosis. In our study, the up-regulation of p53 and phospho-p53 indicated the failure of DNA repair and start a p53-dependent apoptosis, which is in agreement with Gazitt's study [43]. The up-regulation of Bax, an apoptosis-promoting protein, was induced by p53, which is additional evidence of p53-dependent apoptosis [44]. Cell cycle arrest is another result of DNA damage [45]. ATM and ATR are major signals of G2-M phase arrest resulting from DNA damage [46]. The phosphorylation of PI3k and p53 by ATM induces the cell cycle block [47, 48]. The phosphorylation/activation of p53 induces G2/M arrest primarily by disrupting the function of the cyclin B1/cdc2 complex. Specifically, p53 represses cdc25c, a phosphatase that promotes mitosis, after DNA damage [49]. Our finding that cyclin B1 is down-regulated in As_2_O_3_-treated cells is in agreement with those earlier results. By contrast, our finding that cyclin D1 remained unchanged after As_2_O_3_ treatment indicates the absence of obstruction in the transition of G1-S.

Finally, we found that As_2_O_3_ induces a significant dose-related increase in the incidence of cellular senescence. Many factors contribute to the induction of cellular senescence, including telomerase suppression, telomere damage and chromosomal damage, though the main factor is telomere dysfunction [50]. The significant elevations of p53 and p21 that we observed are consistent with cellular senescence. p21 is usually suppressed in malignant cells. The resultant p21 deficiency enables escape from senescence through chromosome doubling, high DNA replication and improved repair potential. In addition, p21 deficiency also decreases the DNA damage checkpoint response (DDR), which is another possible route enabling escape from senescence. As_2_O_3_-induced telomere dysfunction results in p53- and p21-mediated cell apoptosis, G2/M cell cycle arrest and senescence.

In sum, our observations provide new insight into the antitumor effects of As_2_O_3_, which appears to act by interfering with telomerase activity and telomere function, and can perhaps contribute to solving the problem of glioblastoma treatment resistance.

## MATERIALS AND METHODS

### Cell culture and treatment

U87 (human glioblastoma), U251 (human glioblastoma), SHG-44 (human glioma) and C6 (rat glioma) cell lines were obtained from the American Type Culture Collection (ATCC). Rat glioma C6 cells are widely used for *in vitro* experiments. Although they are less malignant than human glioblastoma cells, C6 cells were used as a model to better explain the effect of A_s2_O_3_ on glioblastoma. The cells were grown in Dulbecco's modified eagle medium (DMEM) supplemented with 10% fetal bovine serum (FBS) (Biowest, South America Origin) in a humidified incubator maintained at 37°C with 95% air and 5% CO_2_. As_2_O_3_ (solid state) was purchased from Sigma Chemical Co. (St. Louis, Missouri, USA). After preparing a 5 mM stock solution in phosphate buffered saline (PBS), the solution was filtered and stored at −80°C. The frozen As_2_O_3_ solution is stable for over 6 months. Working concentrations were freshly prepared daily by diluting the stock with serum-free DMEM.

### Cell proliferation assays

The cytotoxicity of As_2_O_3_ toward glioma cells was assessed using MTT assays. Cells in the log growth phase were seeded onto 96-well microplates at a density of 5×10^3^ cells in 200 μl of medium per well and left to attach overnight prior to treatment. As_2_O_3_ was then added to various final concentrations. Dimethyl sulphoxide (DMSO) vehicle served as a control. Twenty microliters of MTT solution (5 mg/ml; Sigma Aldrich, USA) were added 4 h before the end of the incubation period, and the reaction was terminated by adding 10% acidified sodium dodecyl sulfate. Formazan crystals in the cells were dissolved in DMSO, after which the absorbance at 570 nm was measured using a microplate reader (Bio-Tek Instruments, USA).

### Invasion and migration assays

Twenty-four-well plates with BioCoat Invasion Chambers (BD) were used to test the invasion or migration of glioma cells. Each chamber contained an 8-μm-pore polycarbonate transwell membrane, with or without Matrigel coating. Cells (2×10^5^/ml) were re-suspended in 200 μl of serum-free medium and plated on the top side of the membrane without Matrigel for migration assays or with Matrigel for transwell matrix penetration assays. The cells were then incubated at 37°C for 48 h, followed by removal of the cells from the upper chamber with cotton swabs. The migrated and invaded cells on the lower membrane surface were fixed in 4% formaldehyde and stained with 0.1% of crystal violet for 5 min. Five fields of cells were counted randomly in each well under a microscope at 200 x magnification.

### Flow cytometric assays

Glioma cells were plated at 10^5^ cells per well in six-well plates and allowed to adhere for 12 h at 37°C before exposure to As_2_O_3_ solution (0, 2, 4 or 8 μM) for 48 h. To detect cell cycle, collected cells were incubated in 70% ethanol for 12 h at −20°C, washed twice with PBS, and incubated with 1g/mL propidium iodide (PI) and RNase for 25 min. Cell apoptosis was detected using an FITC Annexin V Apoptosis Detection Kit (BD Pharmingen, Inc.). Cells were incubated first in the 1× binding buffer, then for 15 min with PI and FITC Annexin V in binding buffer while shaking. Reactive oxygen species (ROS) were detected using a ROS detection Kit (ZSGB-BIO). The cells were incubated for 30 min in pre-warmed (37°C) PBS containing 1μM CM-H2DCFDA (Molecular Probes, Eugene, OR, USA). The loading buffer was then removed, and the cells were returned to growth medium containing As_2_O_3_ (0, 2, 4, 8 or 16 μM).

### Telomeric repeat amplification protocol assay

Telomerase enzyme activity was measured using a TRAP assay with cell extracts exposed to As_2_O_3_ for 48 h in situ at concentrations of 0, 2, 4, 8 or 16 μM. TRAP assay was performed as previously reported [51]. A TRAPeze kit (Roche Diagnostics) was used to measure the effects of As_2_O_3_ on U87, U251, SHG44 and C6 cell lysates. Total cellular protein (2 μg) was used for each PCR. The PCR products were separated on a PAGE gel.

### Cell senescence staining

Glioma cells were plated at 5×10^4^ cells per well in 6-well plates and exposed to As_2_O_3_ at a concentration of 0, 2, 4 or 8 μM for 2 weeks (the cells were collected for passage on day 7). They were stained with a solution of citric acid, X-gal and ferric iron. Fixed Buffer was used for fixation for 1 h, after which the cells were immersed in cold PBS for observation. Finally, an inverted microscope (Olympus, Japan) was used for photographing.

### Immunoblotting

Immunoblotting was performed as previously reported [51]. Total proteins were extracted from the cultured cells. Samples containing 30-35 μg of total protein were subjected to 8-12% SDS polyacrylamide gel electrophoresis (PAGE), transferred onto a nitrocellulose membrane (Roche), and probed with following monoclonal primary antibodies: anti-actin (Sigma-Aldrich, Inc.), anti-TRF1, anti-hTERT, anti-hTERT^Tyr707^, anti-γ-H_2_AX, anti-53BP1, anti-ATM, anti-p-ATM, anti-ATR, anti-Mer11, anti-Bcl-2, anti-Cyclin B1, anti-Cyclin D1, anti-aurora A, anti-p-aurora A, anti-p53 and anti-p21 (Santa Cruz Biotechnology, Inc.). HRP-conjugated goat anti-rabbit and goat anti-mouse antibody (ZSGB-BIO) were then used as secondary antibodies.

### Immunofluorescence

Immunofluorescence was performed as previously reported [51]. Cells were fixed in 2% formaldehyde and permeabilized in 0.25% Triton X100 in PBS for 5 min at room temperature. For immunolabeling, cells were incubated with primary antibody, washed in PBS and incubated with fluorophore-conjugated secondary antibodies. The following monoclonal primary antibodies were used: anti-TRF1, anti-ATR, anti-γ-H_2_AX, anti-53BP1, anti-Mer11 and anti-hTERT, (Santa Cruz Biotechnology, Inc.). Rhodamine- or DyLightTM488-conjugated goat anti-rabbit and fluorescein- or DyLightTM594-conjugated goat anti-mouse (ZSGB-BIO) served as secondary antibodies. Fluorescence signals were captured using an Olympus Fluoview FV1000 confocal microscope and analyzed using the FV10-ASW 1.6 Viewer program (Olympus, Japan).

### ChIP assays

ChIP assays were performed as previously reported [51] and according to manufacturer's instructions (Millipore). Briefly, cells were fixed in 0.8% paraformaldehyde in 1×PBS, washed extensively in 1×PBS, and lysed in ice-cold lysis buffer (1% SDS, 10 mM EDTA, 50 mM Tris-HCl and protease inhibitors at pH 8.0). The chromatin products were the sonicated (∼100-300 bp) and diluted 10× in dilution buffer (20 mM Tris-HCl, 150 mM NaCl, 2 mM EDTA, protease inhibitors and 1mg/ml BSA at pH 8.0). ChIP was performed using the relevant antibody and captured with Protein A/G-Sepharose. DNA-protein complexes were washed with 1×wash buffer I (20 mM Tris-HCl, 150 mM NaCl, 0.1% SDS, 1% Triton X-100 and 2 mM EDTA at pH 8.0), 2× wash buffer II (20 mM Tris-HCl, 250 mM NaCl, 0.1% SDS, 1% Triton X-100 and 2 mM EDTA at pH 8.0) and eluted in 1% SDS and 100 mM NaHCO_3_. Eluate fractions were de-crosslinked in high-salt solution (200 mM NaCl) at 60°C followed by proteinase K treatment at 50°C. Extracted DNA was subjected to PCR as previously reported [51].

### Statistics

Statistical significance between two treatment groups was evaluated using the Student's *t* test; P < 0.05 was considered significant. SAS version 9.2 (SAS Institute Inc., Cary, North Carolina) and Excel 2013 (Microsoft Corp., Redmond, WA) were used for all statistical analyses.

## SUPPLEMENTARY FIGURE


