# Supplementary material for: Arsenic trioxide inhibits glioma cell growth through induction of telomerase displacement and telomere dysfunction
Source: Oncotarget. 2016 Feb 8;7(11):12682–92. doi: 10.18632/oncotarget.7259 (PMC4914314; doi:10.18632/oncotarget.7259)
Supplement: Supplementary file 1 [file oncotarget-07-12682-s001.pdf]

SUPPLEMENTARY FIGURE

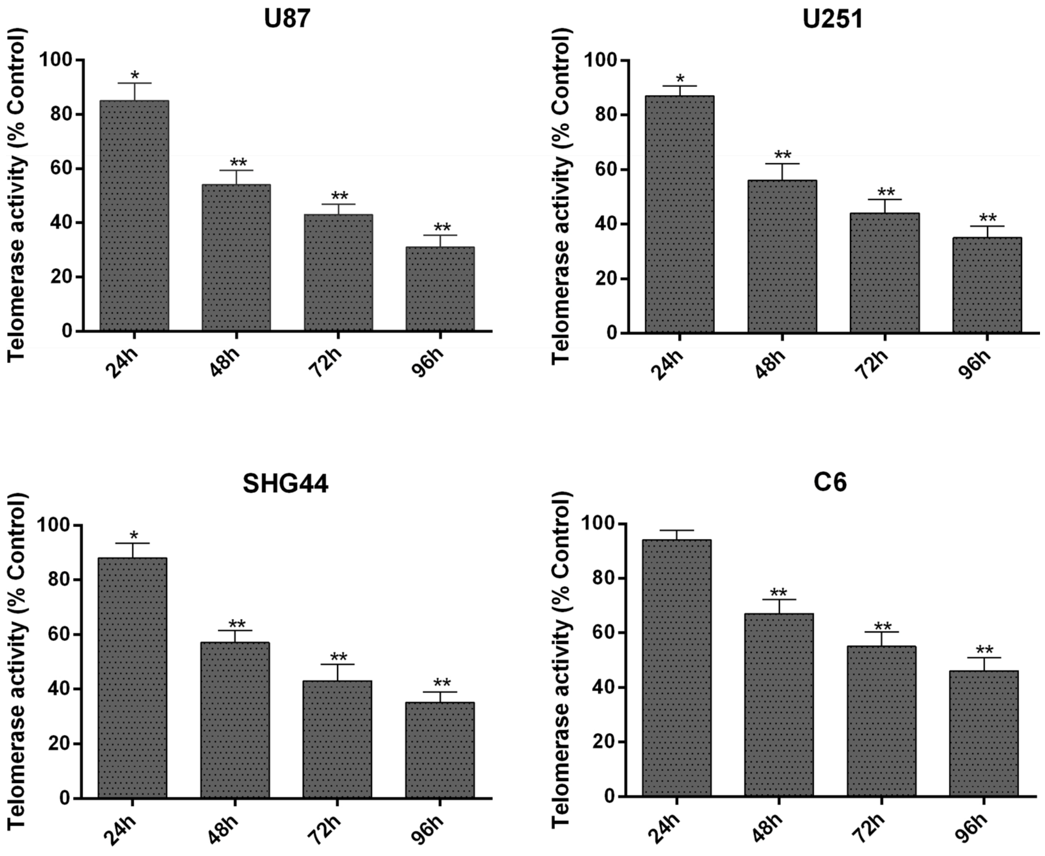

Supplementary Figure S1: Results of TRAP assays showing the telomerase activity in U87, U251, SHG44 and C6 cells after exposure to 4 µM As_2O_3 for 24, 48, 72 or 96 h. Error bars indicate ± s.d., $*P < 0.05$, $**P < 0.01$, two-tailed Student's t-test.
